# Compromising UDP-sugar nucleotide biosynthesis attenuates *Candida albicans* viability, virulence and drug sensitivity^☆^

**DOI:** 10.1016/j.tcsw.2026.100170

**Published:** 2026-02-08

**Authors:** Dhara Malavia-Jones, Ian Leaves, Jemima Onime, Paul O'Neill, Kaizhou Yan, Alistair J.P. Brown, Neil A.R. Gow

**Affiliations:** a MRC Centre for Medical Mycology, University of Exeter, Geoffrey Pope Building, Stocker Road, Exeter EX4 4QD, UK; b Exeter Sequencing Facility, University of Exeter, Geoffrey Pope Building, Stocker Road, Exeter EX4 4QD, UK; c Division of Molecular, Cell and Developmental Biology, School of Life Sciences, University of Dundee, Dundee, UK

**Keywords:** *C. albicans*, Cell wall, Drug target, Sugar nucleotide biosynthesis, Virulence

## Abstract

*Candida albicans* is an opportunistic fungal pathogen that can cause a variety of superficial and life-threatening systemic infections. Relatively few clinically effective antifungal therapies are available, and the increasing prevalence of antifungal drug resistance poses a serious threat in treating these infections. Target validation of biochemical pathways that are essential for fungal growth offers an approach towards the design of novel antifungal drugs that address the growing requirement for new antifungal therapies. Therefore, we used the GRACE library of conditional mutants of *C. albicans* to explore enzymes in the sugar nucleotide biosynthesis pathway as potential drug targets. This pathway provides UDP-*N*-acetylglucosamine (UDP-GlcNAc), UDP-glucose (UDP-Glc) and GDP-mannose (GDP-Man) substrates for the synthesis of the essential cell wall polymers, chitin, β-glucan(s) and mannan(s). We show that the genes encoding GDP-mannose pyrophosphorylase (*SRB1/PSA1/VIG9*), UTP-glucose-1-phosphaturidyl transferase (*UGP1*), phosphoglucose isomerase (*PGI1*) and glucosamine-6-phosphate synthase (*GFA1*) are critical for growth, biofilm formation and virulence in *C. albicans*. Genes encoding other enzymes in the sugar nucleotide biosynthetic pathway (namely *AGM1*, *PMM1*, *PMI1*, *GNA1* and *UAP1*) were not essential for growth but were required for biofilm formation, tissue invasion and virulence. Repression of genes that encode these enzymes also resulted in hypersensitivity to a range of antifungal drugs as well as oxidative and cell wall stressors. These data underline the potential for augmenting antifungal drug development by targeting these enzymes in the treatment of *C. albicans* infections.

## Highlights

• *RB1*, *UGP1*, p *PGI1* and *GFA1* are required for the biosynthesis of sugar nucleotide substrates essential for construction of the fungal cell wall.
• Suppressing growth by repressing genes on the sugar nucleotide pathway did not always correlate with the loss of virulence associated traits.
• Decreased *SRB1*, *UGP1*, *PGI1* and *GFA1* gene expression resulted in hypersensitivity to a range of antifungal drugs and cell wall stressors.

## 1 Introduction

A key limitation in antifungal discovery is the shortage of novel drug targets that can be exploited to combat fungal infections, particularly in the face of rising antifungal resistance (Fisher et al., 2012; Brown et al., 2024; Case et al., 2025; Johnson and Moore, 2025; WHO Antimicrobial Resistance (AMR) Division, 2025). Despite this urgent clinical need, the development of new antifungal agents has lagged significantly behind that of antibacterial therapy, leaving vulnerable populations at risk (Roemer and Krysan, 2014; Hoenigl et al., 2021; Jacobs et al., 2022; WHO, 2025).

Recent estimates suggest that invasive fungal infections, including those caused by *Candida* species, affect over 6.55 million people globally each year, leading to more than 2.5 million attributable deaths (Jacobs et al., 2022; Denning, 2024; WHO Antimicrobial Resistance (AMR) Division, 2025). *Candida* species responsible for superficial and invasive infections include *Candida albicans*, *Candida dubliniensis, Candida parapsilosis, C. metapsilosis* and *C. orthopsilosis* and also related species formerly placed in the *Candida* genus - *Nakaseomyces glabratus (C. glabrata)*, *Pichia kudriavzevii* (*C. krusei*), *Clavispora lusitaniae* (*C. lusitaniae*) and *Candidozyma auris* (*C. auris*) (Denning, 2024; Kullberg and Arendrup, 2015; Talapko et al., 2021; Tamo, 2020). Recurrent vaginal candidiasis, which is predominantly caused by *C. albicans* and *N. glabratus*, affects more than 140 million women (Hashemi et al., 2019; Willems, 2020; Erfaninejad et al., 2022; Denning, 2024). Globally, around 1.6 million cases of invasive candidemia occur per year, causing an estimated 995,000 deaths (Talapko et al., 2021; Denning, 2024; Wolfgruber et al., 2024). These statistics reflect knowledge gaps, inaccuracy and delays in diagnosis, and the limited availability of antifungal treatments with favourable activity spectra and pharmacokinetic profiles (Brown et al., 2012; Revie et al., 2018; Lee et al., 2023; Salmanton-García et al., 2024).

The fungal cell wall is a favoured drug target because it is comprised of polysaccharides and proteins that are not present in humans and because many cell wall components are essential for fungal viability and virulence (Gow, 2025). The essentiality of the cell wall is based on the need for its continuous dynamic synthesis, degradation, and remodelling, to enable a delicate balance of plasticity for growth and rigidification to prevent lysis and maintain viability (Wessels, 1986; Gow and Lenardon, 2023; Gow, 2025). Several classes of antifungal drugs target cell wall biosynthesis, most notably the echinocandins (caspofungin, micafungin, anidulafungin, rezafungin) and ibrexafungerp which inhibit β-(1,3) glucan synthase. More recently fosmanogepix, which is a pro-drug that targets inositol acetyltransferase Gwt1 and consequently GPI-cell wall protein anchoring is under phase 2 clinical trials as a first-in-class broad spectrum antifungal (Jackson et al., 2013; Hoenigl et al., 2021; Gow, 2025). As yet, there are no approved antifungals that target mannan biosynthesis although pradimicin A and pradimicin U, which disrupt mannan biosynthesis are reported to have broad spectrum antifungal activity (Walsh and Giri, 1997; Duangupama et al., 2024; Gow, 2025). Olorofim is another experimental antifungal, first of a new class of antifungal called orotomides that inhibits pyrimidine biosynthesis via inhibition of dihydroorotate dehydrogenase, resulting in cell wall remodelling (du Pré et al., 2020). The polyoxins and nikkomycins inhibit chitin synthases, but these are considered unsuitable for clinical use due to limitations in pharmacokinetics and cellular uptake (Jackson et al., 2013).

The *C. albicans* cell wall has a bilaminate structure consists of an inner, predominantly alkali-insoluble fraction composed of a branched core of β(1,3)- and β(1,6)-glucans, and chitin (β(1,4)-linked *N*-acetylglucosamine), and an outer fibrillar alkali-soluble fraction comprised of mannoproteins with *O*-, *N*- and phospho-linked mannans that are attached to the inner glucan-chitin skeleton via β(1,6)-glucan, which is essential for the formation of the bilaminate wall structure (Gow and Lenardon, 2023; Bekirian et al., 2024; Gow, 2025). The synthesis of the individual components of the wall is coordinated via a series of signalling pathways namely, cell wall remodelling, PKA and Hog pathways (Navarro-García et al., 2005; Munro et al., 2007; Bermejo et al., 2008; Walker et al., 2010; Xiong et al., 2021; Gow and Lenardon, 2023), to ensure that the strength of the wall is sustained even when wall integrity is under assault.

**Fig. 1:**
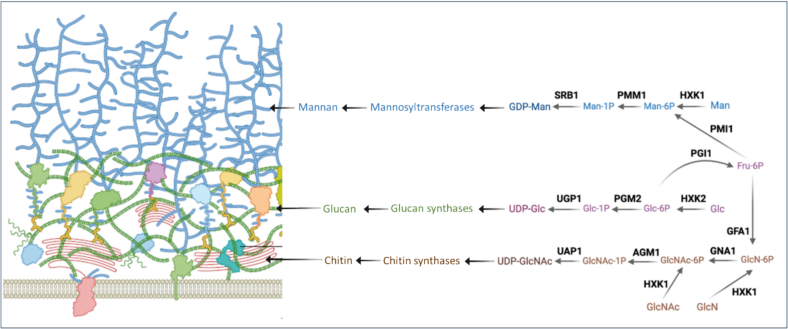
Schematic illustration of enzymes involved in the biosynthesis of GDP-mannose (GDP-Man), UDP-glucose (UDP-Glc) and UDP-*N-*acetylglucosamine (UDP-GlcNAc). These three sugar nucleotides are the substrates for the mannosyltransferases involved in the synthesis of mannan (blue), glucan synthases that make glucans (green) and chitin synthases that make chitin (brown). These represent the major polysaccharides of the cell wall of *C. albicans*. The enzymes are: **Srb1**/Psa1/Vig9 (GDP-mannose pyrophosphorylase; E.C: 2.7.7.13); **Ugp1** (UTP-glucose-1-phosphaturidyltransferase; E.C: 2.7.7.9); **Pgi1** (glucose-6-phosphate isomerase; E.C: 5.3.1.9); **Gfa1** (glucosamine-6-phosphate synthase; E.C: 2.6.1.16); **Hxk1** and **Hxk2** (hexokinase 1; E.C: 2.7.1.59 and 2; E.C: 2.7.1.1); **Agm1** (phosphoacetylglucosamine mutase; E.C: 5.4.2.3), **Gna1** (glucosamine-6-phosphate acetyltransferase; E.C: 2.3.1.4); **Uap1** (UDP-GlcNAc pyrophosphorylase; E.C: 2.7.7.23); **Pgm2** (phosphoglucomutatase E.C: 5.4.2.2) and **Pmm1** (phosphomannomutase; E.C: 5.4.2.8). The cell wall model is reproduced with permission from Gow and Lenardon (2023). (For interpretation of the references to colour in this figure legend, the reader is referred to the web version of this article.)

Chitin, glucan and mannan are all essential for the viability of *C. albicans* and most fungal species (Gow and Lenardon, 2023), and together they comprise approximately 20% of total cell biomass (Klis et al., 2014; Baek et al., 2024). Therefore, the biosynthesis of these cell wall polymers depends on a significant supply of their sugar nucleotide substrates, UDP-glucose, UDP-*N*-acetylglucosamine and GDP-mannose. These three sugar nucleotides are also present in mammalian cells, but their biosynthetic pathways differ from those in fungi. Therefore, we selected nine fungal genes specifically involved in UDP-glucose, UDP-*N*-acetylglucosamine or GDP-mannose production that represent potential drug targets in *C. albicans*. Also, while the human and fungal structures of many of the enzymes in this pathway (Fig. 1) are similar, differences in regions near active site can potentially be exploited for drug development. (Zhou et al., 2022).

Glucose is the main precursor for sugar nucleotides and is converted to glucose-6-phosphate (Glc6P) and fructose-6-phosphate (Fru6P) by hexokinase 2 (HXK2) and phosphoglucose isomerase (Pgi1) (Fig. 1) (Diderich et al., 2001; Green et al., 1988). The role of *C. albicans* Pgi1 likely mirrors that in *S. cerevisiae* and *A. fumigatus* in the conversion of G6P to F6P. However, to date there are no bespoke studies on *C. albicans* Pgi1 to confirm this. Fungi generate UDP-*N*-acetylglucosamine (UDP-GlcNAc) via glucosamine (GlcN) or *N*-acetylglucosamine (GlcNAc), and GDP-mannose via mannose (Man) (Fig. 1).

The essentiality of some genes/enzymes involved in sugar nucleotide biosynthesis has been tested in some fungi. Disruption of *HXK2* in *C. albicans* does not have a strong physiological phenotype, perhaps because hexokinase 1 (*HXK1*) may also phosphorylate GlcN, GlcNAc and Man (Anderson et al., 1978; Boles, HOFMANN and ZIMMERMANN, 1994; Aleshin et al., 1998; Kuser et al., 2008). Crystal structures of Pgi1 from *A. fumigatus*, (Zhou et al., 2022) *S. cerevisiae* (Green et al., 1988) and human (Read et al., 2001) have been generated, but equivalent analysis is lacking in *C. albicans*.

UDP-glucose is essential for cell wall glucan synthesis and is produced by phosphoglucomutase (Pgm2) and UTP-glucose-1-phosphaturidyl transferase (Ugp1 encoded by *UGP1*) (Fig. 1) (Ruiz-Herrera and Ortiz-Castellanos, 2019). *C. albicans* has a single Pgm isoform (encoded by *PGM2*), whilst there are two in *S. cerevisiae* (Boles, Hofmann and Zimmermann, 1994). The crystal structure of Pgm2 has been elucidated in *A. fumigatus* and *C. albicans*, providing insights into its function and raising its potential as a therapeutic target (Yan et al., 2022). *UGP1* deletion is lethal in *S. cerevisiae*, blocking β-glucan synthesis and impairing cell wall integrity (Daran et al., 1997) However, the impact *UGP1* deletion has not been tested in *C. albicans*.

The UDP-GlcNAc pathway is essential for chitin synthesis in *C. albicans* (Munro, 2013). Four enzymes, including glucosamine-6-phosphate synthase (Gfa1 encoded by *GFA1*), catalyse sequential reactions (Fig. 1) to generate UDP-GlcNAc. Gfa1 catalyses the conversion of Fru6P (produced by Pgi1) into GlcN6P which is converted into UDP-GlcNAc by phosphoacetylglucosamine mutase (Agm1), GlcN6P *N*-acetyltransferase (Gna1) and UDP-GlcNAc pyrophosphorylase (Uap1) (Gabriel et al., 2004). Gfa1 catalyses the first committed step in this pathway and is suspected to be essential in *C. albicans* (Smith et al., 1996). Structural analysis of fungal Agm1 underlines its role in the biosynthesis of UDP-GlcNAc (Nishitani et al., 2006; Fang, Du, Raimi, Ramón Hurtado-Guerrero, et al., 2013a; Lockhart et al., 2020)*.* Similarly, the importance of Uap1 and Gna1 for growth and virulence remains to be investigated in *C. albicans*, but its disruption results in an aberrant morphology in *S. cerevisiae* and *A. fumigatus*, (Mio et al., 1998; Fang, Du, Raimi, Ramon Hurtado-Guerrero, et al., 2013b).

GDP-mannose is synthesized by mannose-6-phosphate (Man6P) isomerase (Pmi1), phosphomannomutase (Pmm1) and GDP-Man pyrophosphorylase (Srb1). Pmi1 has been validated as an essential enzyme in *C. albicans*, *S. cerevisiae*, and *A. fumigatus*, and inhibitors that target fungal Pmi1 have been identified (D J Smith et al., 1992a; Cleasby et al., 1996; Fang et al., 2009). *C. albicans PMM1* is functionally homologous to the *S. cerevisiae SEC53* gene, consistent with a likely role in mannose metabolism and protein glycosylation (Smith et al., 1992b). Additionally, Srb1, plays a role in cell wall integrity (Warit et al., 1998). In *C. albicans, SRB1* expression increases in response to oxidative, hyperosmotic and thermal stress (Gao et al., 2019) and is reduced in fluconazole-resistant strains (Ji et al., 2020).

Given that there are gaps in our understanding of the importance of genes involved in sugar nucleotide biosynthesis in *C. albicans,* we undertook a detailed phenotypic characterisation of nine of these genes in relation to growth, pathogenicity, biofilm formation, drug resistance and other virulence traits: *AGM1, GFA1, GNA1, PGI1*, *PMI1*, *PMM1*, *SRB1, UAP1* and *UGP1*. Our findings underline the potential of the sugar nucleotide biosynthesis pathways as promising antifungal drug targets.

## 2 Materials and methods

### 2.1 Microbial strains, growth and media

The GRACE™ strains (Roemer et al., 2003; Xiong et al., 2025) and other *C. albicans* strains used in this study are listed in Supplementary Table S1. All strains were maintained on YPD (1% yeast extract, 2% mycological peptone, 2% dextrose) agar (2%) and grown in YPD broth for 24 h at 30 °C and 200 rpm for experimentation. For transcriptional repression, strains were grown in YPD broth supplemented with 25 μg/ml doxycycline (Dox). Serum Agar (10% human serum, 2% agar) or RPMI supplemented with 10% (*v*/v) human serum and 2% glucose was used to induce filamentation and biofilm formation. Strains were incubated in these media at 37 °C for 24 h or 7 days as indicated.

For growth rate measurement, strains were grown overnight in YPD broth, diluted to an OD_600_ of 0.02 in fresh YPD ± Dox, and 200 μl aliquots added to wells of a Greiner clear flatbottom 96 well plate. Plates were incubated at 30 °C for 24 h and OD_530_ measured every hour using a Tecan plate reader. Growth suppression at 24 h was calculated relative to the control lacking Dox:

$$\mathrm{Suppression}\;\left(\%\right)=\left(1–{\mathrm{OD}}_{\mathrm{Dox}}/{\mathrm{OD}}_{\mathrm{Control}}\right)\times 100$$

$$\mathrm{Range of suppression}=\max\left(\mathrm{suppression}\right)–\min\;\left(\mathrm{suppression}\right)$$

For hyphal development on agar, yeast cells grown overnight in YPD ± Dox broth were washed and adjusted to a concentration of 1 × 10^7^ CFUs/ml and 10 μl of each sample was spotted on to Serum Agar containing different concentrations of glucose and fructose. Plates were incubated at 37 °C for 7 days and photographed. Experiments were performed in triplicate.

### 2.2 Real time RT-PCR

Real time RT-PCR was performed as described in Malavia-Jones et al. (2023). Strains were grown in YPD ± 25 μg/ml Dox for 24 h as described above. Cells were washed three times with sterile distilled water and RNA was extracted using Monarch™ RNA extraction kit (NEB, Catalogue No. T2010S). Samples were then subjected to DNase treatment to remove residual genomic DNA using Monarch RNA purification kit (NEB catalogue No. 2040S). cDNA was synthesized using 500 ng extracted RNA from all samples and M-MLV Reverse Transcriptase (Promega UK, Catalogue No. M1701) as per the manufacturer's specifications. Real time RT-PCR reaction was set up using SYBR™ Green Universal Master Mix (Applied Biosystems, catalogue No. 4309155) as per manufacturer's protocol and RT-PCR reaction was performed on QuantStudio 7 Pro Real Time PCR system (Applied Biosystems). The assay consisted of 10 min denaturation at 95 °C followed by 40 cycles at 95 °C for 15 s and 60 °C for 1 min. Then, melt curve analysis was performed (60 °C to 95 °C at a ramp rate of 0.1 °C/s). Relative expression of target gene was determined by normalising against housekeeping gene, *ACT1* using following formula:

$$\mathrm{\Delta Ct}={\mathrm{Ct}}_{\text{target gene}}–{\mathrm{Ct}}_{\text{housekeeping gene}}$$

$$\text{Relative expression}=2^{-\mathrm{\Delta Ct}}\;2.3$$

### 2.3 Spot assays for drug and stress sensitivity testing

For antifungal susceptibility testing, all strains grown overnight in YPD ± Dox broth were washed and adjusted to 1 × 10^6^ CFU/ml. Cells were then serially diluted 10-fold and 10 μl of each dilution spotted on to YPD ± Dox agar plates containing various antifungal agents. The plates were incubated at 30 °C for 24 h and photographed. Three independent biological replicates were performed.

### 2.4 Biofilm assays

The TT reduction assay was adopted with minor modifications (Ramage et al., 2001). Briefly, biofilms were grown in presence and absence of Dox for 24 h. Mature biofilms were washed twice with PBS and biofilm formation quantified using XTT Cell Proliferation Kit II (Sigma Aldrich, Product No. 11465015001).

### 2.5 In vitro cell damage assay

Human epithelial cells derived from a vulvar squamous cell carcinoma (A-431 cell line; ATCC No.: CRL-1555) were cultured and maintained in DMEM medium supplemented with 10% (*v*/v) heat inactivated foetal calf serum, 5% penicillin and 5% streptomycin. Cultures were incubated at 37 °C, 5% CO_2_ for 4 days to reach confluency. For the assay, cells were seeded in a tissue culture grade clear flat-bottom 96 well plate at 1 × 10^5^ cells/well and further incubated for 48 h to reach 90% confluency. *C. albicans* conditional mutants were grown with and without Dox as described above. The A-431 cells were co-incubated with 2 × 10^5^*C. albicans* cells/well for 24 h at 37 °C and 5% CO_2_. No doxycycline was added to the DMEM medium during co-incubations. Lactate dehydrogenase (LDH) activity was assayed to measure fungal damage to the epithelial cells using the Sigma MAK066 kit. Experiments were performed in triplicate.

### 2.6 Virulence assays in galleria mellonella

Virulence assays were performed in the invertebrate *G. mellonella* wax moth model as described previously with some modifications(Borman et al., 2013). Final (sixth) instar larvae weighing approximately 300 mg each (Galleria Mellonella Research Centre, University of Exeter, Devon, UK) were maintained at room temperature and inoculated within 24 h of receipt. *C. albicans* conditional mutants were grown in YPD ± Dox as previously described and harvested by washing thrice with sterile PBS. Cells were then counted using a Vi-CELL BLU (Beckman) cell counter and adjusted to 2.5 × 10^7^ cells/ml in sterile PBS containing 0 or 50 μg Dox/kg body weight. Individual larvae (*n* = 10 per strain) were inoculated in the left rear proleg with 2.5 × 10^5^ yeast cells (inoculum volume of 10 μl). Control groups of larvae were inoculated with 10 μl of PBS and PBS + 50 μg/kg body weight of Dox. Larvae were then incubated at 37 °C and scored for viability at 24 h intervals. Kaplan-Meier survival plots were generated and statistical differences evaluated using the Mantel-Cox test.

### 2.7 Quantification of cell wall polysaccharides by high-pressure-ion-chromatography (HPIC)

Yeast cells were grown overnight in YPD ± Dox at 30 °C as described above and cell wall polysaccharides analysed as described previously (Mora-Montes et al., 2007). Cells were pelleted by centrifugation, washed twice in water and then sheared using a FastPrep machine (MP Biomedicals). The homogenate was centrifuged at maximum speed (4000 rpm) and the pellet washed with 1 M NaCl to remove soluble proteins. Samples were then heated for 10 min at 100 °C in SDS extraction buffer (500 mM Tris-HCl pH 7.5, 2% (*w*/*v*) SDS, 0.3 M β mercaptoethanol, 1 mM EDTA), before freeze drying. Relative glucan, mannan and chitin levels were determined by quantification of glucose, mannose and glucosamine, respectively, produced by hydrolysis of cell walls with 2 M trifluoroacetic acid at 100 °C for 3 h. Hydrolysates were analysed by HPIC as described by Mora-Montes et al. (2004) with the following modifications. Samples of 0.4 μl were injected into a Dionex carbohydrate analyser equipped with a CarboPac PA20 column (0.4x150mm), guard column and an ED50 Pulsed amperometric detector (PAD). Samples were eluted with a gradient of 5–100 mM at a flow rate of 0.008 ml/min for 25 min. Experiments performed three times independently in triplicate (*n* = 9). One-way ANOVA used for statistical analysis, error bars represent standard errors of mean, p**** < 0.0001.

### 2.8 RNA sequencing and analysis

*C. albicans* conditional mutants (*SRB1*, *UGP1*, *PGI1*, *GFA1* and WT reference) were grown in YPD ± Dox as previously described for 24 h. Total RNA Miniprep Kit (Monarch®, New England Labs, T2010S) was used to extract RNA from the strains. RNA yield and extracted integrity were assayed using Qubit HS Kit and RNA ScreenTape® Analysis respectively. Three biological replicates were obtained for each condition.

RNA sequencing and analysis was performed by the Exeter Sequencing Facility at the University of Exeter. Sequencing was performed using an Illumina NovaSeq6000. Paired end reads were trimmed with fastp version 0.23.1 with a q score trimming of 22 and minimum length of 75. Reads were then aligned to ribosomal database with bowtie v.2–2.3.5 to remove any ribosomal RNA reads. Remaining reads were then aligned to the *C. albicans* genome GCF_000182965.3_ASM18296v3 with STAR v2.7.9a. Genes were quantified with RSEM v1.3.1. Differential gene expression analysis based on the Negative Binomial was determined with DESeq2 v.1.38.3 using default parameters. Gene set enrichment analysis was performed with clusterprofiler v4.6.2 in R. The Benjamin and Hochberg False Discovery Rate (FDR) procedure was used for multiple hypothesis testing correction. To capture broader transcriptional trends, genes with FDR-adjusted *P* value (0.25) showing changes of >2 or < −2 fold were considered to be differentially expressed. KEGG terms for each gene were retrieved from the *C. albicans* database (https://www.genome.jp/kegg-bin/show_organism?menu_type=pathway_maps&org=cal) and GO term annotations were retrieved from the Candida Genome Database (http://www.candidagenome.org/). Volcano plots were generated using EnhancedVolcano v.1.16.0.

## 3 Results

### 3.1 Key genes involved in sugar nucleotide synthesis are important for growth of *C. albicans*

**Fig. 2:**
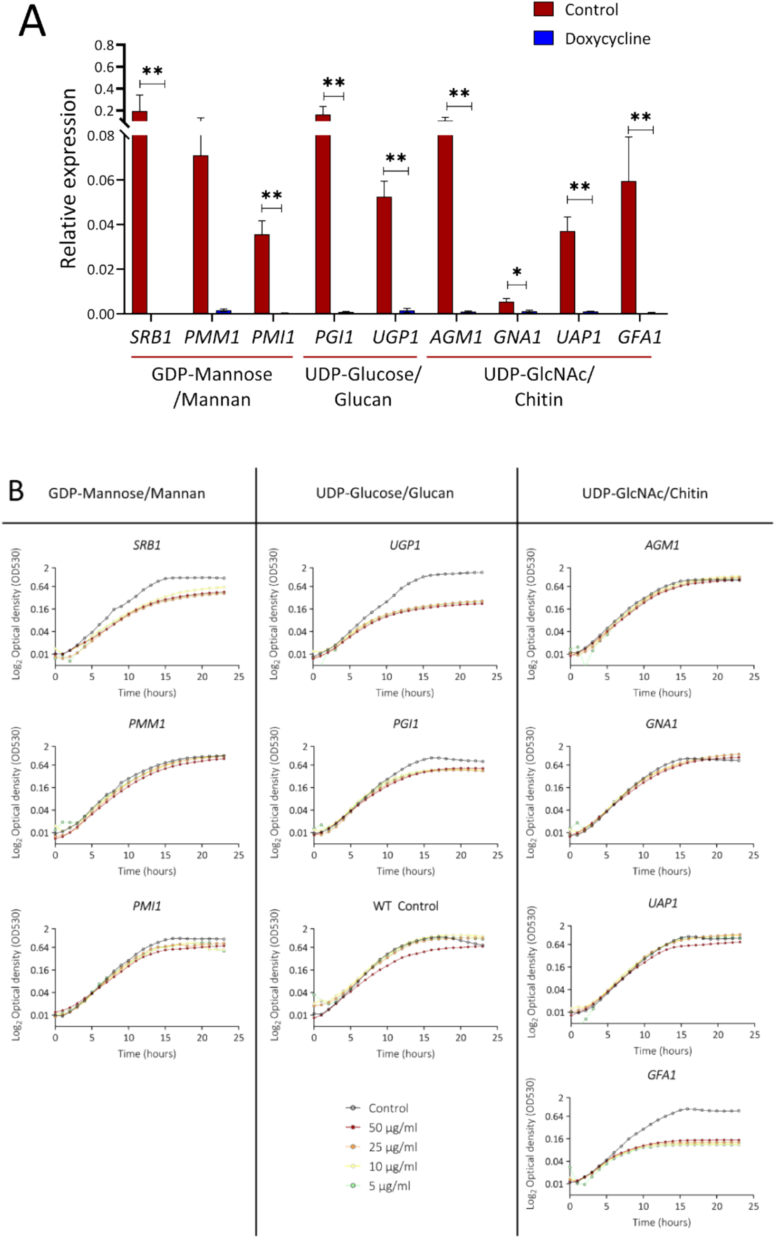
Growth and expression of *C. albicans* GRACE mutants involved in sugar nucleotide biosynthesis. (A) Relative expression of sugar nucleotide genes when grown in YPD in presence (blue bars) and absence (red bars) of 25 μg/ml Dox. Data obtained from two independent experiments performed in triplicates. Student *t*-test used for statistical analysis, error bars represent standard error of mean, p** < 0.01. (B) Mutants were cultured in YPD and YPD containing varying concentrations of doxycycline for 24 h at 30 °C and OD_530_ measured every hour. Repression of *SRB1*, *UGP1*, *PGI1* and *GFA1* inhibited growth of *C. albicans* compared to wild type control (+/-Dox) and compared to respective – Dox controls. Data obtained from three independent experiments performed in triplicate (*n* = 9). B). (For interpretation of the references to colour in this figure legend, the reader is referred to the web version of this article.)

Nine *C. albicans* genes were selected for analysis: *PMI1*, *PMM1* and *SRB1* involved in GDPMan biosynthesis; *PGI1* and *UGP1* involved in UDPGlc biosynthesis; and *AGM1, GFA1, GNA1* and *UAP1* involved in UDPGlcNAc biosynthesis. Using qPCR, we quantified relative expression of target genes when grown in 25 μg/mL Dox for 24 h (Fig. 2A). We observed significant repression of target genes ranging from 3-fold (*GNA1* repression) to 250-fold (*PGI1* repression) suggesting significant suppression of respective enzymes after 24 h growth in Dox.

To evaluate gene essentiality, the growth of the corresponding GRACE conditional mutants and control strains (Table S1) was compared over 24 h in the presence varying doxycycline (Dox) concentrations (0, 5, 10, 25 or 50 μg/mL). Comparable levels of growth suppression were achieved for 5–50 μg/ml of doxycycline for each tested strain. We therefore used 25 μg/mL doxycycline for subsequent experiments. When comparing growth among strains, different degrees of growth suppression were observed for the nine conditional mutants tested (Fig. 2B). Significant growth suppression was achieved for some conditional mutants, most notably *SRB1* (61%), *UGP1* (85%), *PGI1* (43%) and *GFA1* (87%) at 24 h. The *PMI1* mutant exhibited varying degrees of growth inhibition between 5 μg/mL - 50 μg/mL doxycycline, although this effect was less pronounced than for *SRB1*, *UGP1*, *PGI1*, and *GFA1*. While significant transcriptional repression was achieved for all tested conditional mutants, significant growth retardation was only observed for *SRB1*, *UGP1*, *PGI1* and *GFA1.* The lack of growth suppression observed for other conditional mutants, despite the strong transcriptional repression of the target gene by Dox, could have been due to the corresponding enzyme having a high abundance and stability, possibly compounded by a low flux control coefficients with respect to cell wall biosynthesis (Rodaki et al., 2006; Hackett et al., 2016). These factors would mean that cells retain sufficient levels of enzyme to sustain metabolic flux and growth for many hours after transcriptional repression was imposed.

### 3.2 Repression of genes in sugar nucleotide synthesis cause physiological defects

**Fig. 3:**
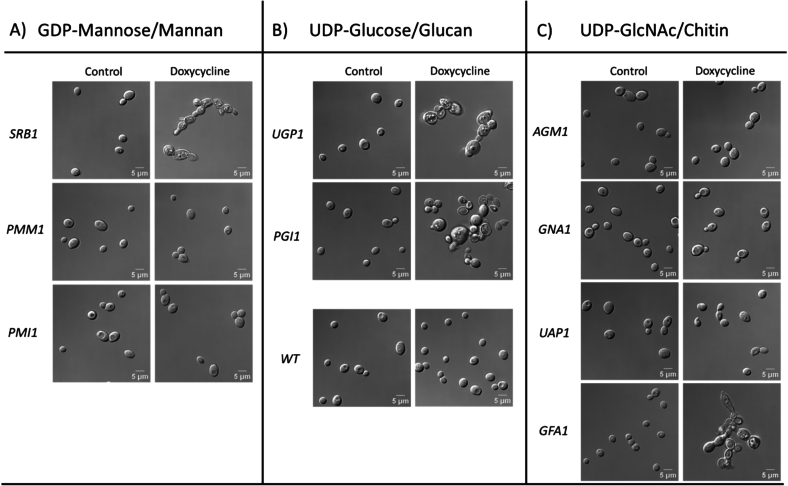
Physiological response of *C. albicans* to repression of enzymes involved in sugar nucleotide biosynthesis. Repression of genes involved in (A) GDP-mannose biosynthesis required for cell wall mannan synthesis, (B) UDP-glucose biosynthesis crucial for cell wall glucan biosynthesis, (C) UDP-*N*-acetylglucosamine biosynthesis which is required for cell wall chitin synthesis. Gene repression achieved by incubating in presence or absence of 25 μg/ml doxycycline for 24 h at 30 °C, 200 rpm. DIC images show that repression of *SRB1*, *UGP1*, *PGI1* and *GFA1* significantly altered the physiology of *C. albicans*. Scale bars represent 5 μm.

The growth of the conditional *SRB1*, *UGP1*, *PGI1* and *GFA1* mutants was significantly inhibited in the presence of Dox compared to the controls, suggesting that compromising the level or activity of *Srb1, Ugp1, Pgi1* or *Gfa1* attenuates the growth of *C. albicans*. This was consistent with the aberrant morphology of the *tet-SRB1*, *tet-UGP1*, *tet-PGI1* and *tet-GFA1* mutants under repressing conditions (Fig. 3), consistent with defects in cell division and separation. *SRB1* is involved in the production of GDP-Man and mannan synthesis, *GFA1* in UDP-GlcNAc production and chitin synthesis, and *UGP1* and *PGI1* in UDP-Glc production and glucan synthesis. The severe growth inhibition coupled with morphological defects observed upon repression of these genes highlighted their potential essentiality.

### 3.3 Repression of sugar-nucleotide biosynthetic genes affects the fungal cell wall and core cellular pathways

**Fig. 4:**
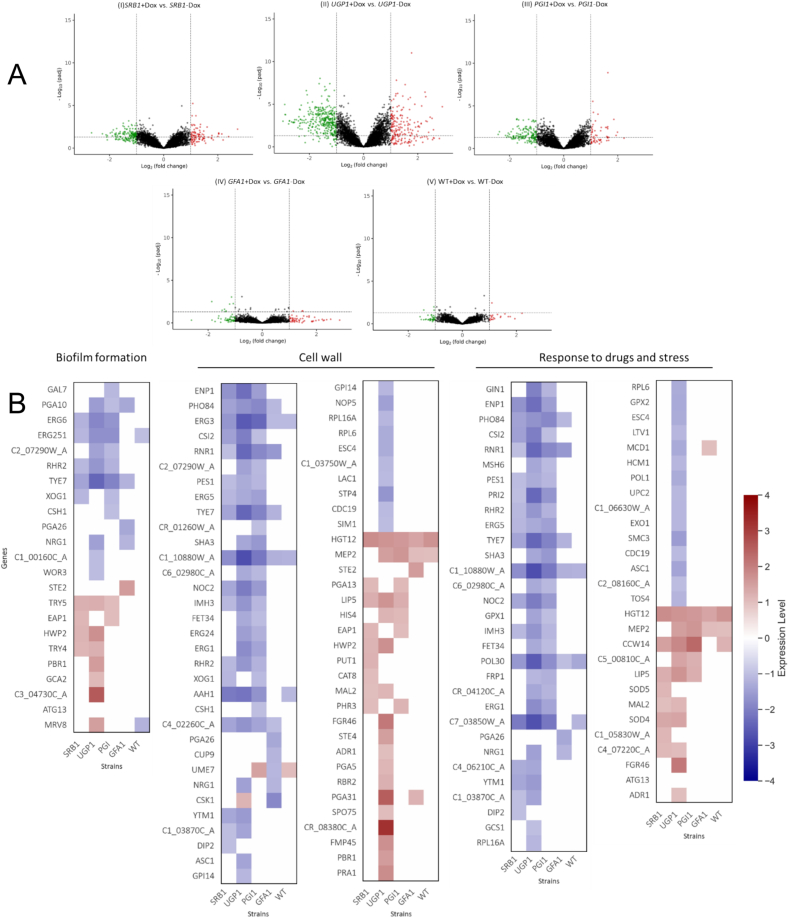

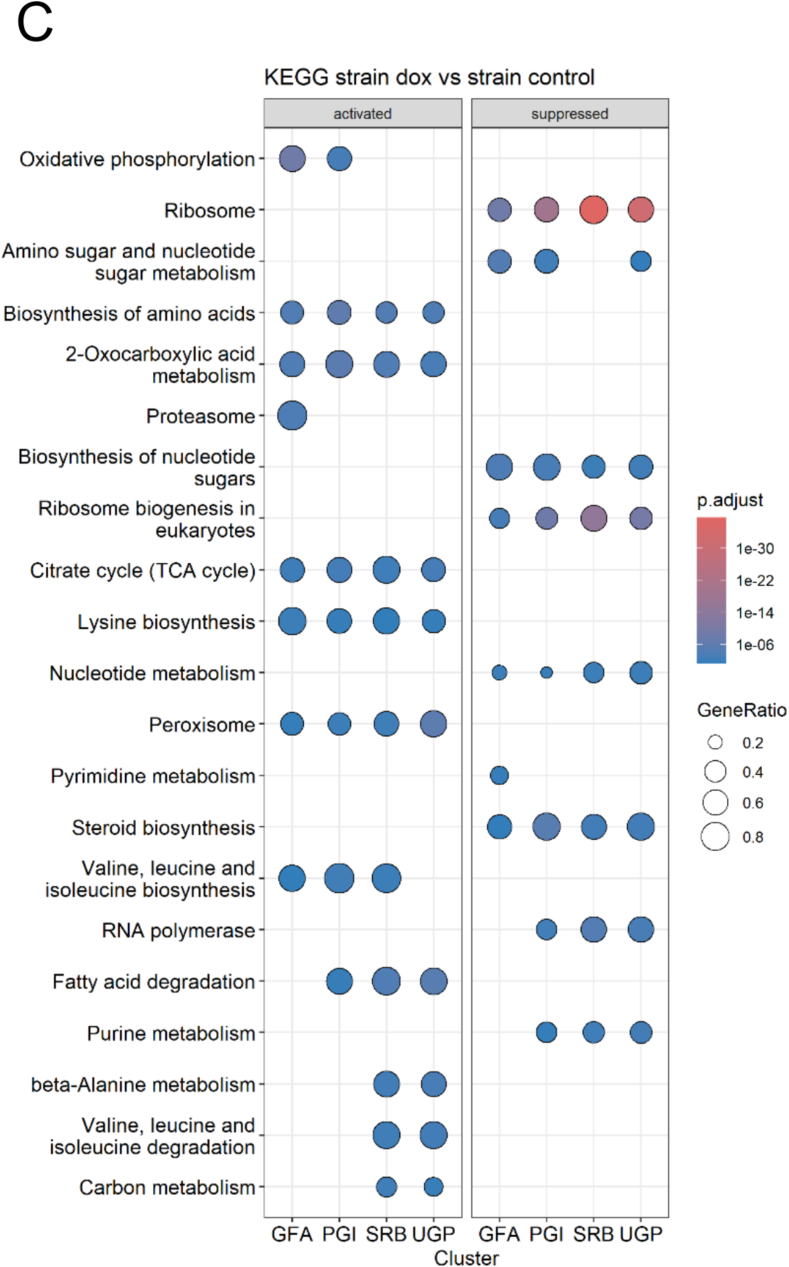
Transcriptional profiles of *SRB1*, *UGP1*, *PGI1* and *GFA1* repressed mutants. (A) Volcano plots of RNA-seq data for (I) *SRB1*, (II) *UGP1*, (III) *PGI1* and (IV) *GFA1* repressed mutants (grown YPD + 25 μg/ml Dox), relative to respective No-Dox controls. Significantly upregulated genes are in red and significantly down-regulated genes are in green. Dotted lines represent the boundary for the fold-change cut-off (Log2 of 0.5). (B) Heat map of genes expressed in *SRB1*, *UGP1*, *PGI1* and *GFA1* repressed mutants with functions in biofilm formation, cell wall proteins and response to various antifungal drugs and stresses. Fold change in gene expression is expressed relative to respective No-dox controls and compared with wild type (WT). (C) KEGG analysis showing activation and suppression of core cellular pathways upon repression of *GFA1*, *PGI1*, *SRB1* and *UGP1* (FDR <0.25, fold change≤ −2 and ≥ 2). (For interpretation of the references to colour in this figure legend, the reader is referred to the web version of this article.)

Next, we investigated the genome-wide transcriptional responses to Dox-mediated repression of *SRB1*, *UGP1*, *PGI1* and *GFA1*. Each conditional mutant and the wild type control (SC5314) was grown in YPD ± 25 μg/ml Dox for 24 h, whereupon cells were harvested for RNA sequencing (Materials & Methods), and subsets of differentially expressed genes identified (statistically significant changes of ≥2-fold relative to the zero Dox control: *n* = 3 independent replicates). A set of 115 genes were differentially expressed in WT + Dox compared to no Dox control. These genes are predicted to play a role in various biological processes including cellular organisation, carbohydrate metabolism, biofilm and cell wall formation (Fig. 4B).

A subset of 108 differentially expressed genes were observed following *GFA1* repression, compared to 301 for *SRB1*, 570 for *UGP1* and 254 for *PGI1* (Fig. 4A). KEGG pathway analyses for all conditional mutants identified several downregulated pathways associated with ribosome biogenesis and function, amino-sugar metabolism, and steroid biosynthesis; and upregulation of the tricarboxylic acid (TCA) cycle, amino acid biosynthesis, and peroxisomal functions. These transcriptional shifts likely reflect growth impairment, oxidative stress and increased energy demands. Significantly, transcriptional changes in genes associated with biofilm formation, cell wall biosynthesis and response to drugs and stresses were observed following repression of *SRB1*, *UGP1*, *PGI1*, and *GFA1* (Fig. 4B).

A subset of genes displayed were differentially expressed in all four mutants: *TYE7* (biofilm formation), *PHO84* (stress response), *RNR1* (cell wall and biofilm regulation), and *C1_10880W_A* (putative filamentous growth) were all down-regulated. *CR_08380C_A* (uncharacterised) and *C3_04730C_A* (uncharacterised) were up-regulated by 4-fold and 2.5-fold in the *UGP1*-repressed strain. Several stress response genes (Fig. 4B) were also down-regulated in the *UGP1*-repressed mutant. Therefore, repression of *SRB1*, *UGP1*, *PGI1*, and *GFA1* elicited transcriptional responses that affected cell wall and core metabolic processes that are likely to influence cell viability and fitness.

### 3.4 Repression of sugar nucleotide biosynthetic genes affects cell wall composition

**Fig. 5:**
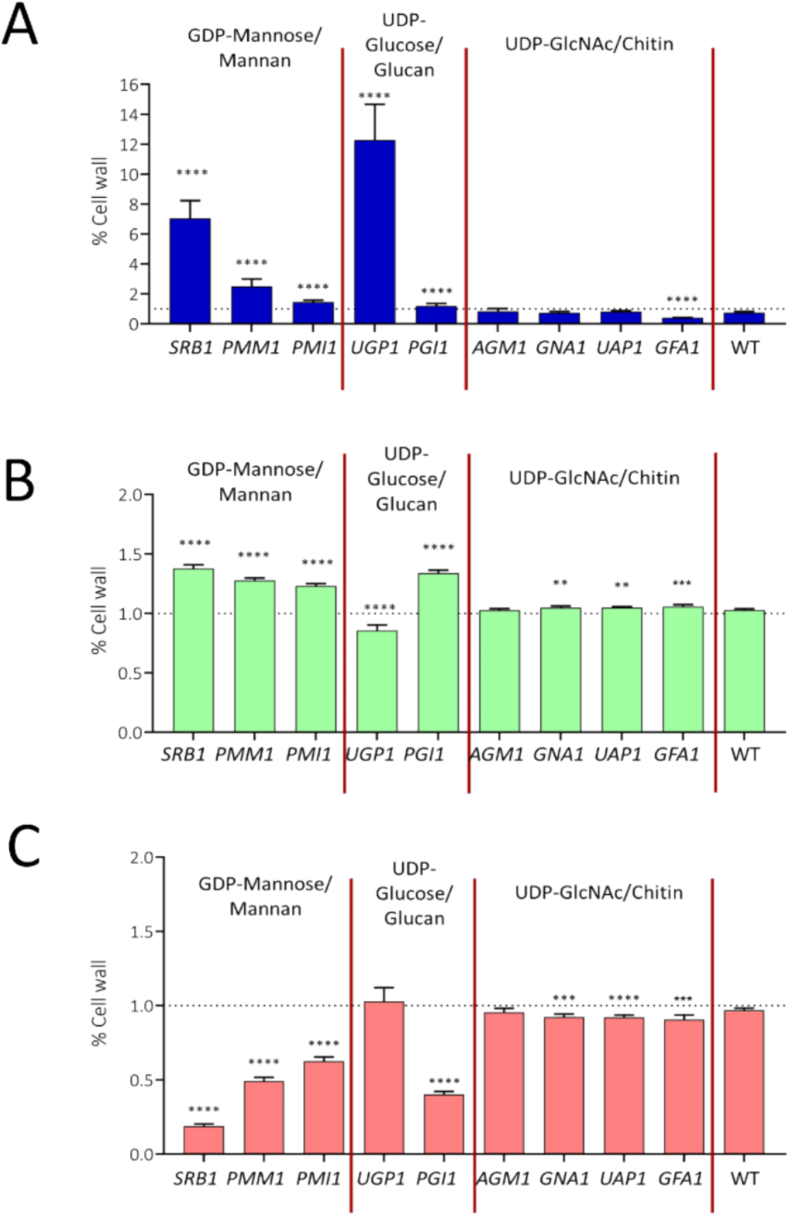
Fold change in relative cell wall polysaccharide (A: chitin, B: glucan, C: mannan) composition in *C. albicans* sugar nucleotide biosynthesis mutants. Cells were grown overnight in YPD (+/− 25 μg/ml Dox) and cell wall was extracted, hydrolysed by Trifluoroacetic acid and polysaccharides in hydrolysates quantified using HPIC. Experiments performed three times independently in triplicate (n = 9). One-way ANOVA used for statistical analysis, error bars represent standard error of mean, p**** < 0.0001.

Repression of sugar nucleotides genes has a direct impact on *C. albicans* cell wall. We therefore quantified changes in overall cell wall composition by comparing levels of cell wall glucan, mannan and chitin in the conditional mutants and wild type strain grown in presence and absence of Dox. Repression of sugar nucleotide biosynthesis genes led to clear changes in the cell wall composition as measured by HPIC (Fig. 5). Repression of genes encoding UDP-mannan synthesis (*SRB1 PMM1* and *PMI1*) exhibited substantial reduction in mannose content relative to wild-type (WT) and no-Dox controls. However, repression of genes involved in chitin (*AGM1*, *GNA1*, *UAP1* and *GFA1*) and glucan synthesis (*UGP1* and *PGI1*) did not lead to substantial changes in chitin and glucan content respectively. For example, the *PGI1*-repressed mutant generated a moderate increase (0.31-fold) in glucan while *UGP1*-repressed mutant showed a modest decrease (0.16-fold) in glucan content (Fig. 5B).

Repression of genes critical for mannan synthesis (*SRB1*, *PMM1* and *PMI1*) also led to significant increases in HPIC-measured glucosamine (Fig. 5A) and glucose (Fig. 5B) levels, suggesting a compensatory relationship between reduced mannan content and increased glucan and chitin. In contrast, repression of genes involved in chitin synthesis (*AGM1*, *GNA1*, *UAP1* and *GFA1*) caused a minor but statistically significant reduction in mannose levels (Fig. 5C) and a modest increase in glucose levels (Fig. 5B) compared to WT control. Substantial increases in cell wall glucosamine (chitin) content were observed when *SRB1* (7-fold change) and *UGP1* (12-fold change) were repressed (Fig. 5A).

### 3.5 Repression of sugar nucleotide biosynthetic genes results in hypersensitivity to cell wall stressors and antifungals

Quantification of cell wall monosaccharides and transcriptional analysis revealed significant alterations in cell wall composition across all nine *C. albicans* conditional mutants, even though only four of the target genes were found to be essential for growth. These changes suggest that perturbation of any one of the three major cell wall biosynthesis pathways may compromise cell wall integrity and cellular fitness, possibly resulting in heightened sensitivity to cell wall-perturbing agents and antifungal compounds.

**Fig. 6:**
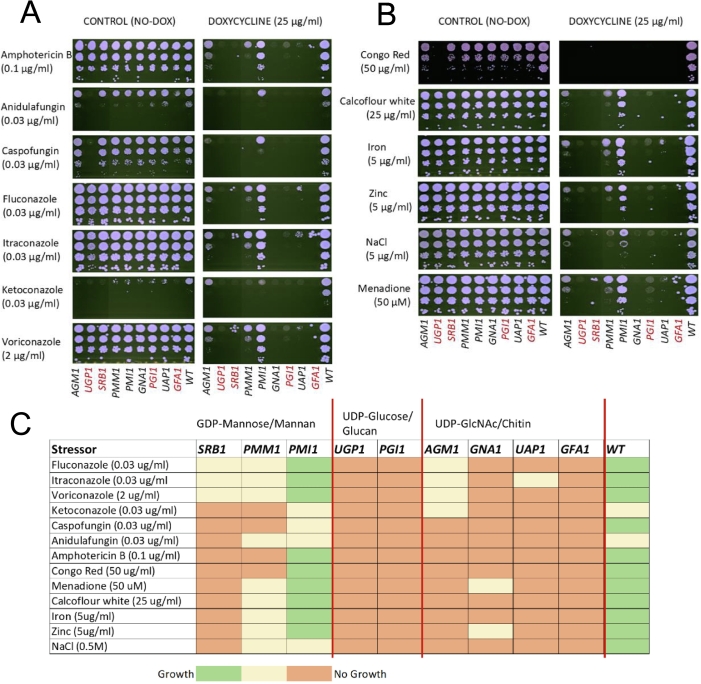
Increased sensitivity to antifungals and various cell wall stresses. Sensitivity of strains to (A) various antifungal drugs and (B) cell walls stressors as tested by spot assay. Genes essential for growth are indicated in red text. (C) Visual representation of sensitivity of repressed strains involved in GDP-mannose/ mannan synthesis, UDP-glucose/ glucan synthesis and UDP-*N*-acetylglucosamine/ chitin synthesis. Red indicates no growth, yellow represents partial growth and green signifies growth comparable to control plates. Serially diluted cells (ten-fold) were spotted on to YPD agar plates with or without 25 μg/ml Dox. Plates were included at 30 °C for 24 h. Representative images taken from three independent experiments performed in duplicate. Growth in absence of antifungal drugs shown in Fig. 2B. (For interpretation of the references to colour in this figure legend, the reader is referred to the web version of this article.)

Spot assays were performed under repressing conditions to evaluate the sensitivity of the mutants to a range of cell wall stressors, antifungal agents, metals, and oxidative stress (Fig. 6A, B) which demonstrated that most conditional mutants exhibited increased sensitivity to all tested compounds. Strains with repressed genes involved in glucan (*UGP1*, *PGI1*) and chitin (*GFA1*, *GNA1* and *UAP1*) synthesis displayed particularly severe stress sensitivities. In contrast, GRACE strains that suppressed *AGM1*, *PMM1*, and *PMI1* demonstrated limited growth defects at low concentrations of stressors.

A summary of the strain-specific sensitivity of responses (Fig. 6C) demonstrates the critical role of sugar nucleotide biosynthesis genes, particularly those involved in glucan and chitin synthesis, in regulating tolerance to antifungal agents, cell wall perturbing compounds, oxidative stress, and metal stressors.

### 3.6 Genes involved in sugar nucleotide biosynthesis are required for filamentation and invasive growth

The yeast-to-hypha morphological transition of *C. albicans* is associated with tissue invasion and necrosis and is associated with substantial remodelling of the cell wall (Mayer et al., 2013; Gow and Lenardon, 2023). Transcriptional analysis of conditional mutants identified genes that were associated with cell wall and biofilm formation (Fig. 4B). Therefore, we investigated how the suppression of sugar nucleotide biosynthetic genes affected filamentation and hyphal growth by growing all strains in RPMI ±25 μg/ml Dox and imaging cells at various time points.

**Fig. 7:**
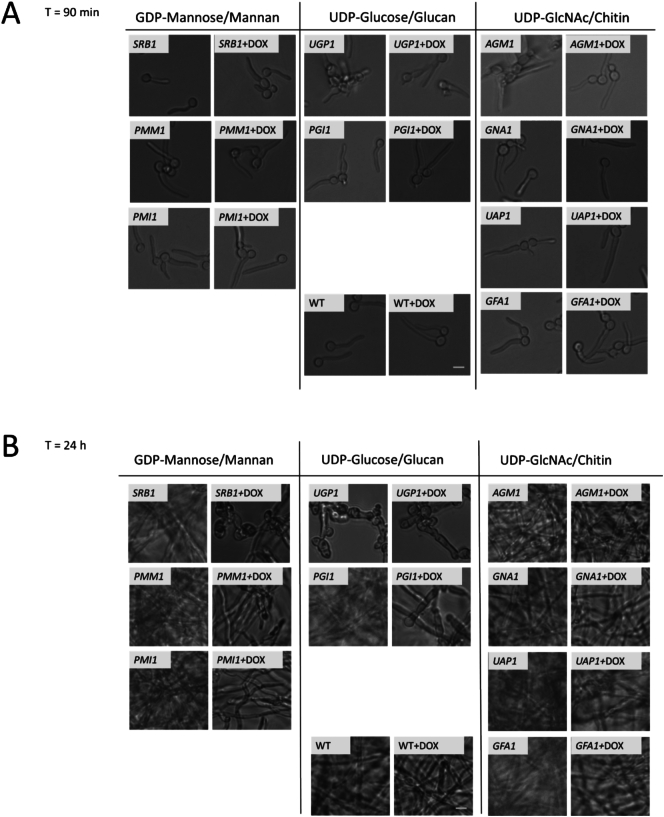

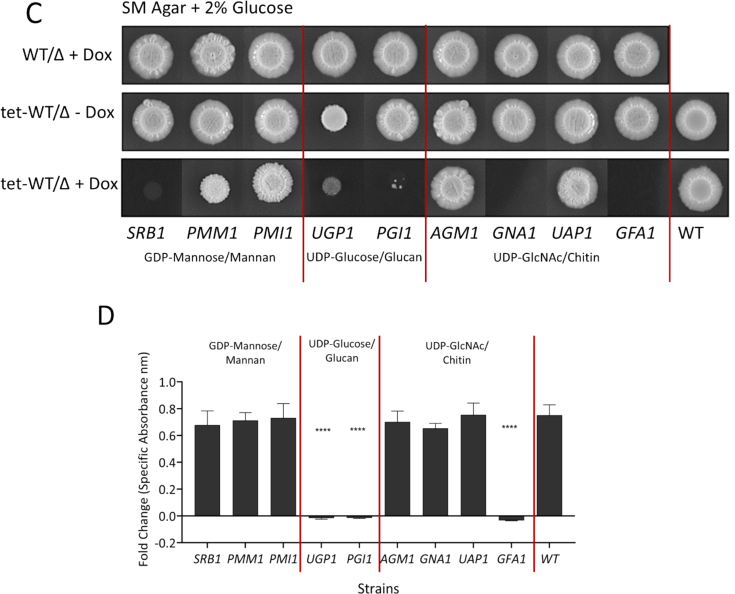
Hyphal induction, filamentation, and biofilm formation in sugar nucleotide biosynthesis mutants. Wild-type (WT) and mutant strains were incubated in RPMI medium at 37 °C for (A) 90 min and (B) 24 h. Filamentous growth in repressed mutants compared to No-Dox and WT controls shows significant defects in repressed mutants at 24 h despite initial hyphal induction at 90 min. Scale bar: 5 μm. Images are representative of three independent experiments. (C) Drop assay showing GRACE (TET-regulated) and DBC mutants with one intact allele (WT) and one deleted allele (Δ) grown on serum containing agar (+ 2% glucose) at 37 °C for 7 days. Representative images from three independent experiments performed in duplicate. (D) XTT assay measuring biofilm formation in the presence and absence of doxycycline (25 μg/ml). The graph depicts fold change (Dox relative to No-Dox) compared to WT control after 24 h. Error bars indicate standard deviation; ****p < 0.0001. Data obtained from three independent experiments performed in triplicate (n = 9).

Between 90 min and 6 h, all nine GRACE strain mutants initiated and established early hyphal induction, with few discernible differences compared to wild-type (WT) and no-Dox controls (Fig. 7A). But by 24 h, significant differences were observed in filamentation in the *SRB1,* and *PGI1* repressed strains compared to WT and no-Dox controls (Fig. 7B). The *GFA1* repressed mutant appeared to maintain filamentous growth, in contrast to previous observations of growth inhibition of yeast cells in YP (Fig. 3).

Filamentous growth was also examined following induction on serum agar (SM) containing 2% glucose. As before, repression of *SRB1*, *UGP1*, *PGI1* and GFA1 severely inhibited growth, and therefore, filamentation at 7 days (Fig. 7C). Consistent with observations in RPMI, *UGP1* No-Dox control displayed filamentation defects in SM agar, although not as severely as that seen in presence of doxycycline. To test this further, we repeated the filamentation experiment using a heterozygous *UGP1/ugp1*Δ mutant from the related DBC collection(Xu et al., 2007). We observed that filamentous growth of this mutant was comparable to the WT control, confirming that the filamentation defects in No-Dox *tet-UGP1/ugp1*Δ control cells stem from suboptimal *UGP1* expression, and that the more severe defects observed in Dox-treated cells were attributable to *UGP1* repression. We conclude that Dox-mediated repression of *SRB1, UGP1, PGI1* or *GFA1* compromise filamentation in RPMI.

Repression of certain sugar nucleotide genes also led to significant defects in biofilm formation and viability. The impact of Dox upon biofilm formation in RPMI was assayed for the conditional mutants and their wild type control. Dox induced dramatic reductions in biofilm formation for the *UGP1*, *PGI1* and *GFA1* strains, but not for the other conditional mutants or the wild type control (Fig. 7D). Although *SRB1* repression resulted in filamentation defects (Fig. 7B), biofilms retained their viability for at least 24 h (Fig. 7D).

### 3.7 Repression of sugar nucleotide genes reduces host cell damage and virulence

**Fig. 8:**
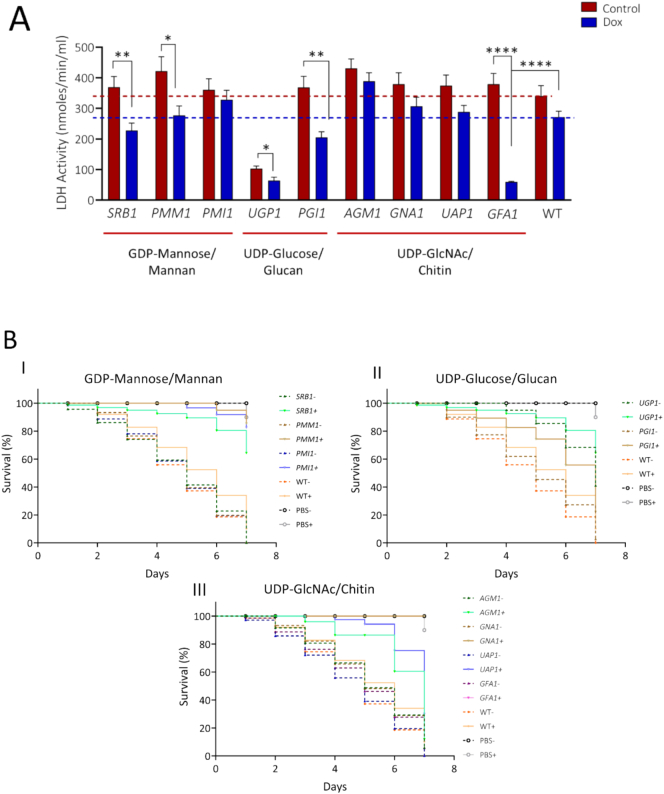
Host cell damage and virulence capacity of mutants in sugar nucleotide biosynthesis. (A) Mutants grown in +/− 25 μg/ml Dox screened for epithelial damage using A-431 cells by LDH assay. The mean LDH released at 24 h post co-incubation is shown for repressed mutants (grown in presence of Dox; blue bars) and No-Dox controls (red bars). Red and blue horizontal lines indicate the mean LDH activity for wild type control (No-Dox) and wild type grown in presence of Dox respectively. Welsh t-test used for statistical analysis; error bars represent standard error of mean; p**** < 0.0001. (B) Survival plots of *G. mellonella* larvae infected with *C. albicans* mutants in: (I) GDP-mannose, (II) UDP-glucose and (III) UDP-*N*-acetylglucosamine biosynthesis in presence (solid lines) and absence (dotted lines) of Dox. No killing or improved survival was observed for a number of repressed mutants. No killing was observed in control larvae injected with equivalent volume of PBS. (For interpretation of the references to colour in this figure legend, the reader is referred to the web version of this article.)

Having observed defects in filamentation in certain conditional mutants, we then determined the impact of repression of sugar nucleotide biosynthesis genes on capacity to damage the host epithelium and virulence. Conditional GRACE strain mutants were grown with and without doxycycline for 24 h and co-incubated with A431 human epithelial cells for an additional 24 h. Fungal damage to the epithelial cells was assessed by assaying the release of lactate dehydrogenase (LDH) from lysed cells. Repression of *SRB1*, *PMM1*, *UGP1*, *PGI1* and *GFA1* resulted in a significant reduction in LDH activity compared to their no-Dox controls, indicating a diminished ability to damage host epithelial cells (Fig. 8A). Notably, *GFA1* repression caused a dramatic decrease in LDH activity, underlining the biofilm and hypha induction deficient phenotype (Fig. 7D). Therefore, sugar nucleotide biosynthesis was critical for the ability of *C. albicans* to damage host epithelial cells.

The invertebrate *Galleria mellonella* model of systemic infection was used to investigate the impact of compromising sugar nucleotide biosynthesis on virulence. *G. mellonella* larvae were infected with the *C. albicans* conditional mutants that had been pre-grown with or without Dox, the larvae incubated with or without Dox, and their survival assessed over time. Survival rates were compared to infections with the WT control strain and the no-Dox controls (Fig. 8B).

Repression of all target genes significantly compromised virulence in this model over a 7-day period (*P* < 0.0001). In contrast, Dox treatment had a minimal effect upon the virulence of the wild type control. Notably, repression of *SRB1*, *PMM1*, *PMI1*, *GNA1*, and *GFA1* markedly improved survival, with 65% to 100% of larvae surviving after day 7. The repression of *PGI1*, *AGM1*, and *UAP1* delayed larval death, with up to 20% of larvae surviving on day 7. Repression of *UGP1* also improved survival, with 60% of larvae surviving to day 7. Interestingly, the no-Dox control for *UGP1* also exhibited elevated survival (40% on day 7), probably because of relatively weak expression from the *tet*-Off promoter even in the absence of Dox.

We conclude that the repression of sugar nucleotide biosynthetic genes significantly attenuated the tissue invasion and virulence of *C. albicans*. This validates these pathways, and in particular SRB, UGP, PGI and GFA as valid drug targets for therapeutic intervention.

## 4 Discussion

The essentiality of the cell wall for growth, viability and resilience under stress makes it a logical target for suppressive chemotherapeutic interventions. The fungal cell wall is composed almost exclusively of molecules that are not found in human tissues and therefore presents a broad selection of possible targets for the development of specific antifungal agents. Targeting key pathways, such as sugar nucleotide biosynthesis, that are required to make all the key carbohydrate components of the cell wall (glucans, chitin, mannans) offers a promising strategy for developing new antifungal agents as they are conserved and essential across most fungal species (Alam et al., 2012; Stalhberger et al., 2014; Ahmadipour et al., 2021; Wijnants et al., 2022; Brauer et al., 2023). The fungal cell wall is also required for protection against external environmental stresses as well as for cellular integrity (Munro et al., 2001; Fuchs and Mylonakis, 2009; Hopke et al., 2018; Wang et al., 2018; Garcia-Rubio et al., 2020).

In this study we explored the essentiality for growth, and hence potential “drugability”, of nine key enzymes specifically involved in the synthesis of core sugar nucleotides, namely GDP-mannose, UDP-glucose and UDP-*N*-acetylglucosamine. We assessed the level of transcriptional suppression that was achievable for each of the GRACE strains by the addition of doxycycline, recognising that this was a non-constant variable between individual conditions and that chemical inhibition of specific drug targets would also not always be capable of complete suppression of enzyme activity. In this context “non-essential” refers to short-term in vitro growth under the tested conditions, and it was evident that repression of several “non-essential” genes resulted in strong virulence defects that in the longer term could significantly compromise the fitness of the fungus in vivo. We observed that some mutants suppressed growth whilst others exerted minor growth defects but displayed attenuated virulence in the *Galleria* wax moth model. We also observed attenuation in the expression of virulence traits such as filamentous growth, biofilm formation, tissue damage and in sensitivity to antifungal drugs. Although the effects of transcriptional repression of the target gene can differ significantly from the impact of pharmacological inhibition of the corresponding enzyme, our data do provide evidence of target vulnerability.

Of these potential targets we show that suppression of the genes encoding GDP-mannose pyrophosphorylase (*SRB1*/*PSA1*/*VIG9*), UTP-1-glucose-phosphaturidyl transferase (*UGP1*), phosphoglucose isomerase (*PGI1*) and glucosamine-6-phosphate synthase (*GFA1*) resulted in the most significant growth inhibition in *C. albicans*. These enzymes also play critical roles in maintaining cell wall integrity and virulence in *S. cerevisiae* and *Aspergillus* species respectively (Aguilera, 1986; Yoda et al., 2000; Lagorce et al., 2002; Zhou et al., 2021, Zhou et al., 2022).

Repressing the expression of certain target genes resulted in significant alterations in cell wall composition, suggesting aberrant synthesis of target polymers and subsequent cell wall remodelling (Fig. 4B and 5). Using HPIC, we quantified mannose, glucose, and glucosamine levels in the cell walls of all conditional mutants. Repression of the *SRB1*, *PMM1* and *PMI1* genes (involved in GDP-mannose biosynthesis) resulted in an expected reduction in mannan content and concomitant increases in glucan and chitin levels, indicating a shift from mannan to glucan and chitin synthesis. The increase in cell wall chitin following *SRB1* or *UGP1* repression might indicate compensatory activation of the hexosamine biosynthesis pathway (Lockhart et al., 2020; Paneque et al., 2023). Under normal conditions, GDP-mannose and UDP-glucose are crucial for glycoprotein and cell wall biosynthesis. When these pathways are disrupted, hexosamine biosynthesis is upregulated, utilizing fructose-6-phosphate and glucosamine to generate glucosamine-6-phosphate (Fig. 1), which is then converted to UDP-GlcNAc (Paneque et al., 2023). This compensatory shift increases UDP-GlcNAc availability, which can result in enhanced incorporation of glucosamine into chitin and elevated cell wall chitin content (Bulik et al., 2003) as observed in our HPIC quantification of cell wall polysaccharides.

The cell wall alterations induced by the repression of sugar-nucleotide biosynthesis genes were observed to have knock on consequences for cellular morphogenesis and invasion. Excess UDP-GlcNAc incorporation into glycoproteins can lead to an imbalance in chitin composition, characterized by increased GlcNAc-modified glycans and reduced mannosylation, ultimately altering the chemical composition of the cell wall (Gow et al., 2017). It is likely that the cell wall changes induced by *SRB1, GFA1* or *UGP1* repression may affect immune interactions and consequently disease progression. Similarly, a reduction in the capacity to cause host cell damage was observed to different degrees following repression of all nine sugar-nucleotide biosynthesis genes studied (Fig. 8A) even though only a subset of these genes caused significant changes in gross cell wall composition (Fig. 5). Interestingly repression of *GFA1*, *UGP1* and *PGI1* severely inhibited biofilm formation (Fig. 7). Repression of *SRB1*, *PMM1* or *PMI1* exerted less severe effects on biofilm formation despite compromising mannan biosynthesis, which is known to play a strong role in cell-cell interactions (Baek et al., 2024). Again, no direct correlation was seen between the effect on growth rate (not greatly affected in *PMM1* and *PMI1* mutants, Fig. 5) and the effect on biofilm formation (Fig. 7).

Alterations in the fungal cell wall can influence antifungal drug sensitivity by altering the target concentration or the permeability to antifungal agents. For example, a reduction in chitin content (as seen in *GFA1* repressed mutant) has been associated with increased susceptibility to echinocandins (Lee et al., 2012; Chung et al., 2025). Similarly, modifications in mannan structures can influence cell wall permeability and affect the binding and efficacy of various antifungal drugs such as amphotericin B (Gow and Hube, 2012; Garcia-Rubio et al., 2020). We observed that the repression of most gene targets caused increased sensitivity to a range of cell wall stressors, antifungal drugs and other stressors. This increased stress susceptibility might be associated with changes to core cellular processes and fitness (Brown, 2023) in addition to a compromised cell wall as suggested by our transcriptional analysis (Fig. 4B and).

The repression of sugar-nucleotide biosynthesis genes also resulted in impaired invasive growth and a diminished ability to cause host damage. In the *G. mellonella* infection model, the repression of genes encoding SRB, UGP, PGI, and GFA had the most severe impact on virulence. However, even the down-regulation of genes encoding PMM, PMI, and GNA, which did not affect growth in vitro, showed a significant attenuation of virulence, with nearly 100% larvae surviving at day 7. This suggests that, while transcriptional repression was not sufficient to block growth over a 24 h period, protracted targeting of these enzymes could significantly reduce pathogenesis.

In summary, we demonstrate that the three pathways leading to the production of sugar-nucleotide precursors of mannan, glucan and chitin biosynthesis are all important for growth in vivo, cellular morphogenesis, host invasion, virulence and drug sensitivity in *C. albicans*. The suppression of growth in the short term does not always correlate with inhibition of virulence associated traits. Target identification has been validated mainly by demonstration of essentiality or importance for growth, however our study also shows that virulence and the capacity for host invasion may be severely compromised even when the target gene product is non-essential.

## CRediT authorship contribution statement

**Dhara Malavia-Jones:** Writing – original draft, Visualization, Validation, Supervision, Software, Methodology, Investigation, Formal analysis, Data curation, Conceptualization. **Ian Leaves:** Writing – review & editing, Validation, Methodology, Investigation, Formal analysis, Data curation. **Jemima Onime:** Writing – review & editing, Software, Methodology, Formal analysis, Data curation. **Paul O'Neill:** Writing – review & editing, Supervision, Software, Methodology. **Kaizhou Yan:** Writing – review & editing, Validation, Methodology. **Alistair J.P. Brown:** Writing – review & editing, Validation, Supervision, Resources, Project administration, Funding acquisition, Formal analysis. **Neil A.R. Gow:** Writing – original draft, Visualization, Validation, Supervision, Resources, Project administration, Investigation, Funding acquisition, Formal analysis, Conceptualization.

## Ethical statement

No animals were used in this study that would be covered under Home Office legislation, UK.

## Declaration of competing interest

The authors declare that they have no known competing financial interests or personal relationships that could have appeared to influence the work reported in this paper.

## Data Availability

Data will be made available on request.

## Appendix A Supplementary data

Supplementary material: Table S1: Strains used in this study. All tetracycline (Tet) regulated strains (tetR) have one deleted allele and one Tet-promoter regulated allele. Corresponding strains from the Double Barcoded Collection (DBC) were also in this study (see text) which have one deleted allele with the second allele intact. Image 1
