# Supplementary material for: Compromising UDP-sugar nucleotide biosynthesis attenuates Candida albicans viability, virulence and drug sensitivity
Source: Cell Surf. 2026 Feb 8;15:100170. doi: 10.1016/j.tcsw.2026.100170 (PMC12915214; doi:10.1016/j.tcsw.2026.100170)
Supplement: Supplementary file 1 — Supplementary material: Table S1: Strains used in this study. All tetracycline (Tet) regulated strains (tetR) have one deleted allele and one Tet-promoter regulated allele. Corresponding strains from the Double Barcoded Collection (DBC) were also in this study (see text) which have one deleted allele with the second allele intact. [file mmc1.docx]

**Supplementary files:**

| Gene name | ORF ID | Genotype | Reference |
| --- | --- | --- | --- |
| *SRB1* | orf19.6190 | *srb1::his3::hisG/his3::hisG leu2::tetRGAL4AD-URA3/LEU2* | (Roemer *et al.*, 2003) |
| *PMM1* | orf19.2937 | *pmm1::his3::hisG/his3::hisG leu2::tetRGAL4AD-URA3/LEU2* |  |
| *PMI1* | orf19.1390 | *pmi1::his3::hisG/his3::hisG leu2::tetRGAL4AD-URA3/LEU2* |  |
| *UGP1* | orf19.1738 | *ugp1::his3::hisG/his3::hisG leu2::tetRGAL4AD-URA3/LEU2* |  |
| *PGI1* | orf19.3888 | *pgi1::his3::hisG/his3::hisG leu2::tetRGAL4AD-URA3/LEU2* |  |
| *AGM1* | orf19.5013 | *agm1::his3::hisG/his3::hisG leu2::tetRGAL4AD-URA3/LEU2* |  |
| *GNA1* | orf19.837 | *gna1::his3::hisG/his3::hisG leu2::tetRGAL4AD-URA3/LEU2* |  |
| *UAP1* | orf19.4265 | *uap1::his3::hisG/his3::hisG leu2::tetRGAL4AD-URA3/LEU2* |  |
| *GFA1* | orf19.1618 | *gfa1::his3::hisG/his3::hisG leu2::tetRGAL4AD-URA3/LEU2* |  |
| SC5314 |  | *his3::hisG/his3::hisG leu2::tetRGAL4AD-URA3/LEU2* |  |

Table S1: Strains used in this study. All tetracycline (Tet) regulated strains (tetR) have one deleted allele and one Tet-promoter regulated allele. Corresponding strains from the Double Barcoded Collection (DBC) were also in this study (see text) which have one deleted allele with the second allele intact.
